# Trabectedin for L-Type Sarcoma: A Retrospective Multicenter Study

**DOI:** 10.3390/curroncol31110502

**Published:** 2024-11-01

**Authors:** Sercan Ön, Barış Köksal, Zafer Arık, Burcu Caner, Duygu Ercan Uzundal, Ozan Yazıcı, Burcu Arslan Benli, Eda Eylemer Mocan, Can Cangür, Zeynep Gülsüm Güç, Seval Akay, Merve Keskinkılıç, Hande Dik Avcı, Burçak Karaca Yayla, Burcu Çakar, Ulus Ali Şanlı

**Affiliations:** 1 Department of Medical Oncology, Tepecik Training and Research Hospital, 35180 Izmir, Türkiye; 2 Department of Oncology, Hacettepe University Cancer Institute, 06410 Ankara, Türkiye; dr_bariskoksal87@hotmail.com (B.K.); drzaferarik@gmail.com (Z.A.); 3 Department of Oncology, Atatürk Government Hospital, 09020 Aydın, Türkiye; drburcucaner@gmail.com; 4 Department of Oncology, Gazi University Medical School Hospital, 06560 Ankara, Türkiye; duyguercan89@gmail.com (D.E.U.); drozanyazici@gmail.com (O.Y.); 5 Department of Oncology, Adana City Hospital, 1370 Adana, Türkiye; brcarslan51@gmail.com; 6 Department of Oncology, Ankara University Medical School Hospital, 06620 Ankara, Türkiye; edaeylemer@gmail.com; 7 Department of Oncology, Selçuk University Medical School Hospital, 42130 Konya, Türkiye; ccangur@yahoo.com; 8 Department of Oncology, Katip Çelebi University Atatürk Training and Research Hospital, 35150 Izmir, Türkiye; zeynepgsevgen@hotmail.com; 9 Department of Oncology, Izmir City Hospital, 35540 Izmir, Türkiye; drsevalakay@hotmail.com; 10 Department of Oncology, Burdur Government Hospital, 15000 Burdur, Türkiye; mervekeskinkilic90@gmail.com; 11 Department of Internal Medicine, Ege University Medical School Hospital, 35100 Izmir, Türkiye; handedik@hotmail.com; 12 Department of Medical Oncology, Ege University Tilay Aktaş Oncology Hospital, 35100 Izmir, Türkiye; karacaburcak@hotmail.com (B.K.Y.); burcu.cakar@gmail.com (B.Ç.); ulus.ali.sanli@ege.edu.tr (U.A.Ş.)

**Keywords:** liposarcomas, leiomyosarcomas, ecteinascidin 743, trabectedin, drug toxicity, targeted radiation therapy

## Abstract

(1) Background: Metastatic L-type sarcomas (liposarcoma and leiomyosarcoma) are rare and have a poor prognosis. Trabectedin is an effective agent that can be used after anthracyclines. This study was designed to evaluate the real-life effectiveness and safety of trabectedin. (2) Methods: A retrospective multicenter study was conducted on patients who were treated with trabectedin for metastatic L-type sarcomas at ten tertiary oncology centers between 2015 and 2023. The objective response rate (ORR), disease control rate (DCR), time to treatment failure (TTF), and overall survival (OS) were evaluated in the cohort. Cox regression analysis was used to determine prognostic factors for survival. (3) Results: A total of 98 patients (52% liposarcoma and 48% leiomyosarcoma) were included in the study. The median treatment line was three (range: 1 to 6). Thirteen patients (13.3%) underwent local treatment due to oligoprogression, and dose reduction was required in seventeen patients (17.3%) due to toxicity. The ORR and DCR were 16% and 42%, respectively. The median TTF was 3 months, and the median OS was 10 months. In univariate analysis, a significantly longer median TTF was observed in patients who underwent local treatment (*p* = 0.008), obtained objective responses (*p* < 0.001), and underwent dose reduction (*p* = 0.002). No statistical differences were observed according to the histologic subtype and metastatic site. In the multivariate analysis for OS, it was found that obtaining an objective response was a good prognostic factor (*p* = 0.003), while the presence of liver metastases was associated with a poor prognosis (*p* = 0.016). (4) Conclusion: Trabectedin is a suitable option for L-type sarcoma after doxorubicin-based treatments. Survival was not worse in patients who underwent dose reduction. The use of local therapies simultaneously with trabectedin can be effective.

## 1. Introduction

Soft tissue sarcomas (STSs) are heterogeneous, mesenchymal tumors that account for less than 1% of all cancers [1]. Sarcomas have more than 50 histopathologic subgroups due to the high differentiation capacity of mesenchymal cells [2]. Liposarcomas (LPSs) and leiomyosarcomas (LMSs) are the most common mesenchymal tumors, accounting for 15% and 11% of all adult STSs. Despite their histology, biology, and clinical behavior heterogeneity, LPSs and LMSs are collectively referred to as L-type sarcomas in the literature [3]. LPSs originate from adipocytes and are most commonly localized in the extremities and retroperitoneum. They have four main histologic subgroups: well-differentiated, dedifferentiated, myxoid/round cell (MLPS), and pleomorphic liposarcoma. LMSs originate from smooth muscle or their mesenchymal precursor. They are most commonly localized in the extremities but can also be localized in the smooth muscle of large vessels, the gastrointestinal tract, and the uterus [2]. These anatomical and histologic subgroups have different clinical courses and prognosis [4,5].

The majority of patients are in the early stage, while approximately 10% are in the metastatic stage at the time of diagnosis. In the early stage of the disease, metastasis develops in approximately 25% of patients after treatments [6,7]. In the advanced stage of the disease, the prognosis is poor, with patients typically having a median survival of 12 to 15 months [8,9]. According to the current ESMO-EURACAN-GENTURIS Soft Tissue and Visceral Sarcoma Clinical Practice Guidelines, doxorubicin, either as a single agent or in combination, is recommended as the first-line treatment in the metastatic period and is the standard in our clinical practice [10]. Trabectedin, eribulin, pazopanib (for non-liposarcoma tumors), and a gemcitabine–docetaxel combination are treatment options for patients progressing or who are ineligible for anthracycline.

Trabectedin is a semi-synthetically produced anti-tumor drug derived from Ecteinascidia turbinata. It binds to guanine at the N2 position in the minor groove of DNA. This binding causes DNA to bend towards the major groove, inhibiting DNA replication and transcription. Consequently, this delay in the cell cycle results in cellular cytotoxicity [11]. In the treatment of metastatic L-type sarcoma, it yielded 4.2 months of progression-free survival (PFS) and 12.4 months of overall survival (OS) compared to dacarbazine in the post-anthracycline period, demonstrated its efficacy, and entered clinical practice with this indication [12]. The drug is licensed in Turkey for similar indications. Prospective studies are essential in establishing standard treatment options in oncology, yet there is an ongoing need for additional data on rare tumors such as sarcomas. Retrospective, real-life data, including diverse patient populations, offer valuable insights that contribute significantly to our daily clinical practice. These insights are essential in enhancing our understanding and management of these less common malignancies. Due to the limited data available in our country, we conducted a retrospective multicenter study to assess the effectiveness and safety of trabectedin in treating metastatic L-type sarcomas.

## 2. Materials and Methods

This retrospective multicenter study is based on a review of hospital medical records of patients treated with trabectedin for metastatic L-type sarcomas between 2015 and 2023 at ten tertiary oncology centers across Turkey. The eligibility criteria were as follows: aged 18 years or older, treatment with trabectedin for L-type sarcomas, and receipt of at least two cycles of trabectedin. All patients had either previously received anthracycline for metastatic disease, were ineligible for anthracycline, or had completed the cumulative dose of anthracycline in the adjuvant period, per the indications for trabectedin use in our country.

Treatment response and disease progression were assessed through clinical and radiological evaluation by the treating physician, using the RECIST 1.1 (Response Evaluation Criteria for Solid Tumors) criteria. The objective response rate (ORR) was calculated as the percentage of patients whose tumor regressed (partial response—PR) or disappeared (complete response—CR) while on trabectedin. The disease control rate (DCR) represented the percentage of patients with CR, PR, or stable disease. The time to treatment failure (TTF) was defined as the length of time between the initiation of trabectedin treatment and the permanent discontinuation of trabectedin due to objective tumor progression, toxicity, or death. Overall survival (OS) was defined as the time between the initiation of trabectedin treatment and death or last follow-up. Safety was evaluated based on the Common Terminology Criteria for Adverse Events (CTCAE) v5.0.

Statistical analyses were performed using the Statistical Package for Social Science 27 program (SPSS, Inc., Chicago, IL, USA). Mean and standard deviation are presented for normally distributed continuous variables, while median and minimum-maximum values are given for non-normally distributed continuous variables. The frequency and percentages for categorical variables have been provided. The chi-squared, Fisher’s exact, and Mann–Whitney U tests were used to compare the relationship between clinicopathological factors and survival. The Kaplan–Meier method was utilized to estimate the time to treatment failure and overall survival. The data for patients lost to follow-up were definitively censored at the time of their last contact. Univariate and multivariate Cox regression analysis were utilized to identify prognostic factors for survival. The study utilized the 95% confidence interval (CI) to evaluate the association between survival time and each independent factor. All *p*-values were two-sided, and those with a value below 0.05 were deemed statistically significant.

## 3. Results

### 3.1. Patient Characteristics

The study included 98 patients from ten centers who received trabectedin treatment for metastatic L-type sarcomas between 2015 and 2023. Patient characteristics are given in Table 1. To summarize briefly, the mean age was 54.5 years (SD +/− 12.1), and 54% of the patients were female. The Eastern Cooperative Oncology Group (ECOG) performance status was good (PS 0/1) in 88 patients (90%). The number of patients with leiomyosarcomas (52%) and liposarcomas (48%) was comparable. The majority of the patients (64.3%) had primaries located in the retroperitoneum and abdomen. Recurrent disease with distant metastasis occurred after primary surgery, representing 72.4% of cases. Additionally, 27.6% of patients were presented with de novo metastatic disease. The most common site of metastasis was the lung (68.4%), followed by the liver (23.4%) and bone (15%).

### 3.2. Trabectedin Exposure and Local Treatment

Patients received trabectedin as the median third-line treatment (range: 1–6). A total of 44% of patients received trabectedin in the first or second line and 30% in the third line. One patient received trabectedin in the sixth line. The trabectedin dosage was reduced in 17 (17.3%) patients due to toxicity. Fourteen patients (14.3%) were deemed ineligible for anthracycline treatment, forty-five patients (45.9%) experienced disease progression while on anthracycline, and thirty-nine patients (39.8%) reached the maximum allowable cumulative dose of anthracycline during the adjuvant period. A total of 13 patients (13.3%) underwent local treatment for metastases due to oligoprogression while on trabectedin. The most common local treatment was radiotherapy (RT) (60%); other patients (40%) had surgery. RT was administered concurrently with trabectedin. In five patients who had surgery, the median time between the last dose of trabectedin and surgery was 4 weeks (range: 3–7 weeks). Of the patients with oligoprogression following an objective response, 37.5% received a local treatment. In contrast, of the patients with oligoprogression without prior objective response, only 8.5% received a local treatment (*p* = 0.002).

### 3.3. Response to Treatment

Among all patients, four achieved a complete response (4.1%), twelve had a partial response (12.2%), twenty-five had stable disease (25.5%), and fifty-seven had a progressive response (58.2%). The ORR and DCR were 16% and 42%, respectively. Four patients who experienced complete responses received 6, 8, 16, and 34 cycles of trabectedin, respectively. In one patient, treatment was continued beyond complete response. In the other three patients who had a CR, treatment was discontinued. Two of these patients had a durable CR and were monitored without further intervention, while one patient subsequently progressed and thus resumed trabectedin treatment. In the MLPS subgroup, CR was achieved in three out of sixteen patients. Among MLPS patients, the ORR was 37.5% and the DCR was 50%. The ORR was significantly higher than the other histology (non-MLPS L-type sarcomas) (*p* = 0.017). The ORR in uterine leiomyosarcoma was lower at 7.1%, while in non-uterine leiomyosarcoma, it was 17%, but the difference was not statistically significant (*p* = 0.39). Moreover, whether trabectedin treatment was given in the first two lines or later did not make a difference in the ORR (18% and 14.5%, respectively).

### 3.4. Time to Treatment Failure

**Figure 1:**
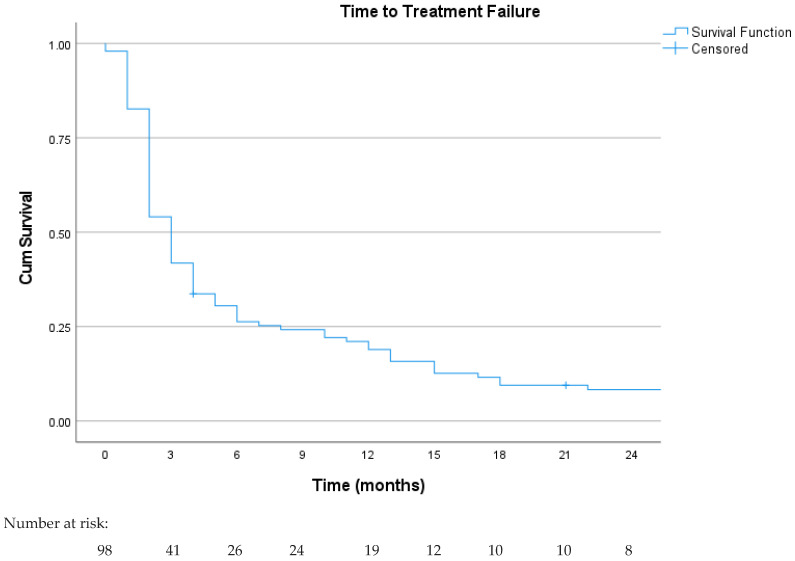
Kaplan–Meier curve for time to treatment failure in the overall study population.

**Figure 2:**
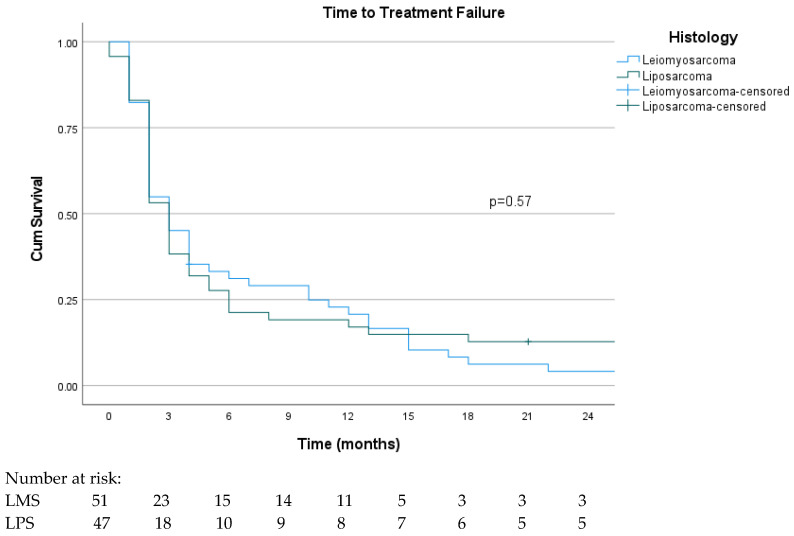
Kaplan–Meier curve for time to treatment failure according to histologic subtype.

The median follow-up was 10 months, and the median TTF was 3 months (95% CI: 2.5–3.5), as shown in Figure 1. The 6-month TTF was 26.5%, and the 12-month TTF was 19.4%. In patients who underwent local treatment for metastasis, the median TTF was 15 months (95% CI: 1.8–28.2), which was statistically longer in these patients (*p* = 0.008). The TTF curve was consistent across histological subtypes, and the median TTF was 3 months for both (LMS vs. LPS), as shown in Figure 2. Despite the improved response rates in patients with MLPSs, the TTF remained at 3 months (95% CI: 0.9–5.6). In univariate analysis, a significantly longer median TTF was observed in patients who obtained objective responses (*p* < 0.001) and dose reduction due to toxicity (*p* = 0.002). No statistical differences were observed according to the trabectedin line (first and second line or beyond the second line *p* = 0.47), metastatic site (*p* = 0.075), and performance status (*p* = 0.24). In the multivariate analysis for the TTF, objective response (*p* = 0.010), local treatment (*p* = 0.038), and dose reduction due to toxicity (*p* = 0.043) were found to be good prognostic factors.

### 3.5. Overall Survival

**Figure 3:**
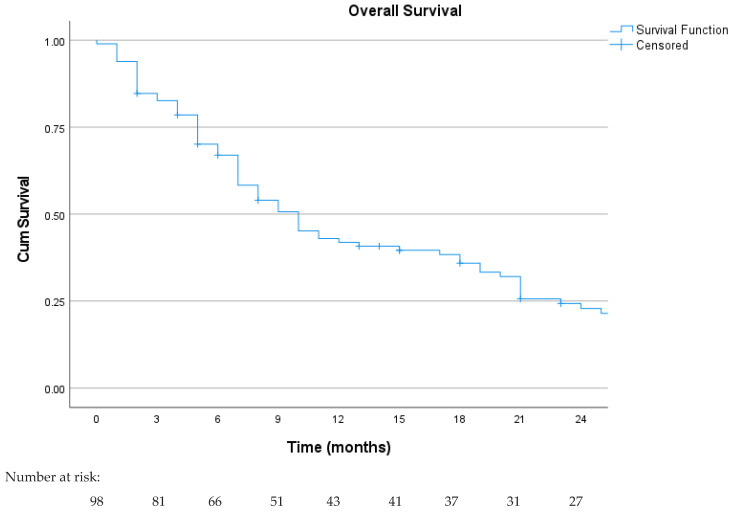
Kaplan–Meier curve for overall survival in the overall study population.

**Figure 4:**
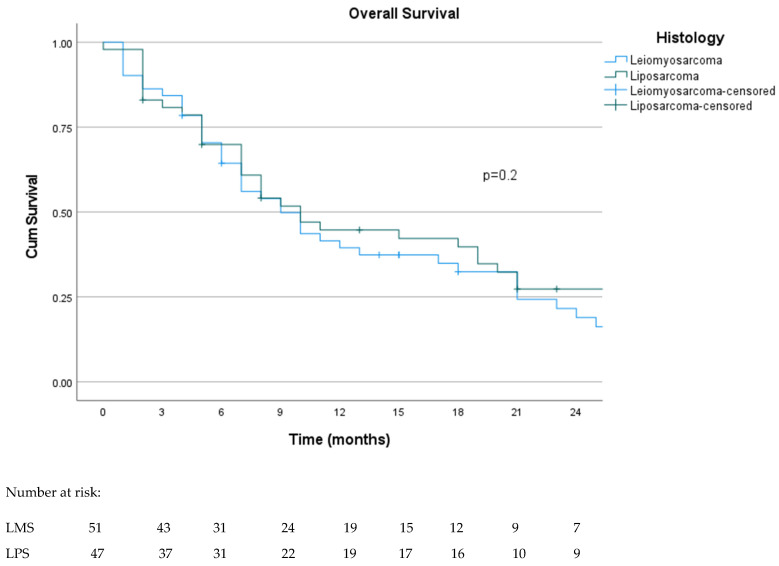
Kaplan–Meier curve for overall survival according to histologic subtype.

The median OS was 10 months (95% CI: 7.4–12.6), as shown in Figure 3. The 18-month and 36-month survival rates were 37.4% and 10%, respectively. The median OS was similar between histology: 9 months (95% CI: 5.6–12.3) for LMS and 10 months (95% CI: 6.3–13.6) for LPS (*p* = 0.2) (see Figure 4). The 18-month survival in MLPS patients was 42%, significantly better than other histological subtypes (*p* < 0.05), but the median OS was 10 months (95% CI: 0.1–24.3). Patients who underwent local treatment of metastases and achieved an objective response had a statistically significantly longer OS in univariate analysis (*p* = 0.013 and <0.001, respectively). Additionally, patients who underwent dose reduction due to toxicity had a slightly improved OS of 19 months (95% CI: 8.4–29.4), although it did not reach statistical significance (*p* = 0.05). Patients with liver metastases had a shorter OS (*p* = 0.005). No statistical differences in OS were observed according to the performance status (*p* = 0.16) and trabectedin line (*p* = 0.16). The multivariate analysis for OS found that objective response was a good prognostic factor (*p* = 0.003), while the presence of liver metastases was associated with a poor prognosis (*p* = 0.016).

### 3.6. Toxicity

The most frequent adverse events were hematologic (see Table 2). Anemia was observed in 67.3% of the patients, neutropenia in 36.7%, and thrombocytopenia in 32.7%. Most of them were low-grade and tolerable. Primary G-CSF prophylaxis was administered to 55% of the patients. However, nine patients developed neutropenic fever. Notably, three of the patients who received primary prophylaxis experienced neutropenic fever. Transaminase elevation was the second most common (55%) toxicity. A low grade of creatinine increase was observed in six patients (6.1%) and did not cause drug discontinuation or dose reduction. The treatment led to dose reduction in seventeen patients due to adverse effects, including thrombocytopenia in six patients (6.1%), transaminitis in five patients (5.1%), neutropenia or neutropenic fever in four patients (4.1%), and anemia in two patients (2.1%). In one patient, the drug was permanently discontinued due to rhabdomyolysis.

## 4. Discussion

In the current study, the TTF was set as one of the endpoints to evaluate the efficacy of concurrent local treatments with trabectedin instead of progression-free survival. The management of metastatic disease in oncology practice involves using local therapies such as radiotherapy, ablation, and surgery for oligometastatic or oligoprogressive disease. Due to this consideration, the TTF may better represent real life than progression-free survival. Retrospective studies have shown that local therapies in treating metastatic sarcomas can provide long-term survival, especially in the oligometastatic patient group [13,14,15]. According to international guidelines, local therapies alone or combined with systemic therapy in the presence of oligometastatic disease are among the most recommended treatment options [10].

Upon reviewing the literature, the efficacy and safety of concurrent radiotherapy with trabectedin were tested in a non-randomized phase I/II study. In that study, metastatic/unresectable L-type and non-L-type sarcomas were included, and radiotherapy was administered to patients after the first cycle of trabectedin without waiting for the progression. In the phase II arm, which included 26 patients, the ORR was 70%, the median PFS was 9.5 months, and the median OS was not reached during the 14-month follow-up period [16]. Our analysis showed that the median TTF-receiving local treatment was notably extended to 15 months. While it was a good prognostic factor in the univariate analysis for OS, this effect disappeared in the multivariate analysis. The reason for this was that the presence of an objective response was also included in the analysis. Local treatments were applied more frequently in patients who achieved objective responses. This can guide us in terms of patient selection. There may be an increased risk of toxicity when administering concomitant therapy. The prospective study mentioned above suggested that trabectedin could potentially raise the risk of radiotherapy-associated pneumonitis. However, no cases of pneumonitis were observed in our patients who underwent radiotherapy. In our study, 40% of patients underwent surgery as a local treatment, trabectedin treatment was continued after surgery, and the toxicity profile was not different from that of the general population. The limited patient population locally treated due to oligoprogression underscores the significance of our study, which demonstrates the efficacy and safety of concurrent local treatments with trabectedin. These findings are particularly important in tumor groups with limited treatment options and short response times.

In prospective phase II/III studies, a median PFS of 3.2 months–4.2 months and a median OS of 13.9 months–12.4 months were found with trabectedin in patients with metastatic L-type sarcomas [12,17]. Although the TTF endpoint was used in our study, it was found to be three months shorter than that of prospective studies. Similarly, OS was 10 months shorter than in prospective studies. In the phase III study by Demetri et al., similar to our study, more than half of the patients received trabectedin as a third line or later. The majority of the patients had LMSs, and there was no information provided about the locations of the tumors [12]. Moreover, due to the retrospective nature of our study, patients with unknown ECOG performance status and those with an ECOG performance status of two were included (10%). Performance status, availability, accessibility of trabectedin, and other therapeutic options before and post-systemic treatment could affect results. Considering all these factors, making a comparative assessment can be quite challenging. It is crucial to consider these factors when evaluating the study results thoroughly.

The lungs are the most common and first site of metastasis in soft-tissue sarcomas [18]. The true incidence of liver metastases in L-type sarcomas is unknown due to the rarity and heterogeneity of sarcomas. In leiomyosarcoma and retroperitoneal sarcomas, the lung remains the most common site of metastasis, while they have a higher propensity to metastasize to the liver than other sarcomas [19]. The presence of liver metastases is an independent poor prognostic factor in metastatic sarcomas [20]. In the present study, it was found that the lung was the most common site for metastasis. Additionally, liver metastasis was observed in a quarter of the patients. It was noted that although the presence of liver metastasis did not affect the TTF, it was identified as a poor prognostic factor for OS in both univariate and multivariate analyses.

Trabectedin mostly provides disease stabilization, with relatively low objective response rates. In our study, the overall objective response rate in the entire population was 16.3%, consistent with both prospective and retrospective real-life data [12,17,21,22]. The nationwide retrospective TrObs study in Italy revealed that the ORR for L-type sarcomas treated with trabectedin was 16.6%. L-type sarcomas showed better response rates compared to other histological types [23]. In both studies, not surprisingly, the patients with an objective response obtained a significantly longer TTF and OS than those who did not. MLPSs are an aggressive subtype with distinct molecular and clinical features [24]. They are known to be chemosensitive in contrast to other soft tissue sarcomas. Trabectedin appears to be more effective in both MLPSs and translocation-associated sarcomas due to its unique mechanism of action, which involves binding trabectedin to DNA. In the MLPS subgroup, the ORR varies between 15 and 51% [25]. In the present study, consistent with the literature, the ORR (37.5%) and complete response rate (18.75%) were significantly better in the MLPS patient group. Despite a higher proportion of patients in this group living beyond one year, the TTF and OS were comparable to those of other histology. It is essential to acknowledge that the findings may be affected by the relatively small sample size of patients in the study.

In early phase studies, the optimal dose of trabectedin was determined as 1.5 mg/m^2^ 24 h infusion, which was approved in Europe and our country [17,26,27]. However, in different countries, such as Japan, lower doses are used due to racial differences in the pharmacokinetic properties of the drug [28,29]. In the prospective non-interventional YON-SAR study, 41% of patients had trabectedin treatment initiated at a clinician-decided dose lower than 1.5 mg/m^2^. Dose reduction was performed in 48% of patients due to toxicity. There was no difference in the effectiveness between patients who received a reduced dose and those who later underwent dose reduction. However, the drug’s efficacy in patients who developed toxicity was not compared with that in patients who did not [22]. In the TrObs study, over half of the patients received a dose of trabectedin at 1.3 mg/m^2^. Response rates and PFS were similar between the two dose groups. Moreover, patients who started with a reduced dose had a longer OS [23]. In our study, the TTF was longer in patients with dose reductions due to toxicity. Although OS was numerically better, this improvement did not reach statistical significance. The relationship between toxicity and efficacy has been demonstrated for immune checkpoint inhibitors and tyrosine kinase inhibitors [30,31]. Trabectedin has various effects on immune cells within the tumor microenvironment. Its mechanism of action, particularly on mononuclear phagocytes, has not been fully understood [32]. Considering all of this, while it is incorrect to generalize the results obtained from a retrospective study with a small number of patients, we believe these data are valuable as they have not been previously shown in the literature. However, this conclusion needs to be supported by more extensive studies.

Trabectedin has a favorable toxicity profile even in heavily pretreated patients. In the phase III study, the usual clinical side effects were nausea (73%) and fatigue (67%). The widespread laboratory abnormalities were transaminase elevation (45%), neutropenia (49%), and anemia (39%). Frequently, side effects are low-grade, but elevated transaminase (25%) and neutropenia (37%) are the most common high-grade (≥grade 3) side effects. The dose reduction rate due to toxicity was 35% [12]. Due to the retrospective nature of our study, clinical side effects such as fatigue and nausea were reported insufficiently. Hematologic side effects and transaminitis were the most common side effects, consistent with the literature. Anemia was the most common hematologic side effect, which may have been disease-related or due to previous systemic therapies. Despite primary GCSF prophylaxis in 55% of patients, neutropenia was observed in 36.7%. In one patient, the drug was permanently discontinued due to rhabdomyolysis. Rhabdomyolysis is a potentially fatal side effect, mainly in the first two cycles, fortunately occurring in less than 1% [33]. The toxicity profile of trabectedin was consistent with previous studies and was tolerable.

The study has some limitations due to its retrospective nature and the small number of patients, resulting from the rarity of L-type sarcomas. The safety analysis may be compromised due to the inclusion of patients who have received at least two courses of trabectedin. The statistical analysis could not include tumor grade because of insufficient pathology reports, despite its known impact on survival outcomes based on the existing literature [20]. The results of this study must be verified by prospective studies with a larger patient population.

## 5. Conclusions

In conclusion, our data showed that in L-type sarcomas, trabectedin seems to be a suitable option after doxorubicin-based treatments with generally well-managed side effects, survival was not worse in patients who underwent dose reduction, and the TTF was even better in this group. However, the number of patients was small. Using local therapies simultaneously with trabectedin can be safe and effective. However, it should be evaluated case-by-case in this specific tumor group, where treatment options are limited. Since sarcomas generally have a poor prognosis, the combined use of single-agent treatments with proven effectiveness in the first step is being evaluated. In a recent study, doxorubicin plus trabectedin combination in first-line therapy achieved superior PFS and OS in patients with metastatic or unresectable leiomyosarcomas compared with doxorubicin alone [34]. Studies may soon move trabectedin to the first step in L-type sarcomas, considering the effectiveness of the combination in the new leiomyosarcoma study.

## Figures and Tables

**Table 1:** Patient Characteristics.

Age, Years (Mean)	54.5 (SD ± 12.1)
Gender	
Female	53 (54.1%)
Male	45 (45.9%)
Performance status	
ECOG 0–1	88 (89.8%)
ECOG 2 or unknown	10 (10.2%)
Histology	
Leiomyosarcoma	51 (52%)
Uterine	28 (28.6%)
Non-uterine	23 (23.5%)
Liposarcoma	47 (48%)
MLPS	16 (16.3%)
Dedifferentiated LPS	15 (15.3%)
Well-differentiated LPS	7 (7.1%)
Pleomorphic LPS	6 (6.1%)
Site of primary tumors	
Retroperitoneal/intra-abdominal	63 (64.3%)
Extremity/trunk	32 (32.7%)
Other	3 (3%)
Trabectedin indication	
Progression under the anthracycline	45 (45.9%)
Received adjuvant anthracycline	39 (39.8%)
Anthracycline ineligible	14 (14.3%)
No. of the line of trabectedin	
≤2 line	43 (43.9%)
>2 line	55 (56.1%)
Local treatment for metastasis	
Yes	13 (%13.3)
No	85 (%86.7)

LPS—liposarcoma, MLPS—myxoid liposarcoma.

**Table 2:** Common adverse events.

Adverse Events	All grades *n* (%)	Grade 3 *n* (%)	Grade 4 *n* (%)
Anemia	66 (67.3%)	9 (9.2)	-
Neutropenia	36 (36.7%)	8 (8.2%)	8 (8.2%)
Trombocytopenia	32 (32.7%)	7 (7.1%)	5 (5.1%)
Febrile neutropenia	9 (9.1%)	9 (9.1%)	-
Transaminitis	54 (55.1%)	10 (10.2%)	3 (3.1%)
Nausea	19 (19.4%)		
Fatigue	10 (10.2%)	-	-
Anorexia	8 (8.1%)	-	-
Constipation	6 (6.1%)	-	-
Creatinine increase	6 (6.1%)	-	-
Rhabdomyolysis	1 (1.1%)	1 (1.1%)	-

## Data Availability

The datasets generated during the current study are available from the corresponding author upon reasonable request.
